# When three is not a crowd: trispecific antibodies for enhanced cancer immunotherapy

**DOI:** 10.7150/thno.81494

**Published:** 2023-01-22

**Authors:** Antonio Tapia-Galisteo, Marta Compte, Luis Álvarez-Vallina, Laura Sanz

**Affiliations:** 1 Immuno-oncology and Immunotherapy Group, Biomedical Research Institute Hospital 12 de Octubre, Madrid, Spain.; 2 Cancer Immunotherapy Unit (UNICA), Hospital Universitario 12 de Octubre, Madrid, Spain.; 3 H12O-CNIO Cancer Immunotherapy Clinical Research Unit, Spanish National Cancer Research Centre (CNIO), Madrid, Spain.; 4 Department of Antibody Engineering, Leadartis S.L., Madrid, Spain.; 5 Molecular Immunology Unit, Biomedical Research Institute Hospital Universitario Puerta de Hierro Majadahonda, Madrid, Spain.

**Keywords:** antibody engineering, cancer immunotherapy, trispecific antibody, T cell engager, NK cell engager

## Abstract

Despite the clinical success of the first bispecific antibody approved by the FDA against B cell malignancies (blinatumomab), many obstacles remain such as dosing, treatment resistance, and modest efficacy in solid tumors. To overcome these limitations, considerable efforts have been dedicated to the development of multispecific antibodies, opening up new avenues to address both the complex biology of cancer and the onset of anti-tumoral immune responses. Simultaneous targeting of two tumor-associated antigens is presumed to enhance cancer cell selectivity and reduce immune escape. Co-engagement of CD3, along with agonists of co-stimulatory molecules or antagonists of co-inhibitory immune checkpoint receptors in a single molecule, may revert T cell exhaustion. Similarly, targeting of two activating receptors in NK cells may improve their cytotoxic potency. And these are only examples of the potential of antibody-based molecular entities engaging three (or more) relevant targets. From the perspective of health care costs, multispecific antibodies are appealing, since a similar (or superior) therapeutic effect could be obtained with a single therapeutic agent as with a combination of different monoclonal antibodies. Despite challenges in production, multispecific antibodies are endowed with unprecedented properties, which may render them more potent biologics for cancer therapy.

## Introduction

In 2021, the 100^th^ antibody-based product was approved by the FDA 1. Approximately half of the antibodies (Ab) in the market are indicated for the treatment of oncological patients, confirming the key role of antibodies in cancer drug discovery. Most of them are conventional monospecific bivalent IgG, but seven are bispecific antibodies (BsAb), four of them approved in 2022, and another two are currently in review. As of March 2019, the commercial clinical pipeline included over 85 BsAb and ~86% were being tested in patients with cancer 2. These data reflect the growing interest in the field of multispecific antibodies.

The first BsAb was described by Nisonoff *et al.* in early 1960s, based on the combination of binding fragments from two different polyclonal sera through mild re-oxidation 3. The advent of monoclonal Ab (mAb) technology allowed the development of BsAb obtained from quadromas (hybrid-hybridomas) 4,5 or based on the chemical conjugation of Fab fragments obtained after pepsinization of hybridoma-derived mAb 6,7. However, it was the recombinant DNA technology along with antibody engineering which prompted the eclosion of a thriving field, with a plethora of different formats 8, resembling or not native IgG. In fact, the first BsAb approved by the FDA for clinical use in 2014, blinatumomab, lacks an Fc region. As an evolution of BsAb, trispecific antibodies (TsAb) have experienced a sudden interest in the last few years. However, the proof of concept was provided more than 30 years ago, using conjugation of three Fab to obtain a molecule able to engage effector T cells with two different arms, and tumor cells with a third one 9,10. As of Nov 2022, around 20 molecular entities with three (or even four) specificities are in early phase clinical trials for the treatment of hematological malignancies or solid tumors. This review outlines the fundamental strategies used for the generation of multispecific Ab, describes recent advances and emerging applications and provides future perspectives for this field.

## Building blocks for the design of multispecific antibodies

**Figure 1:**
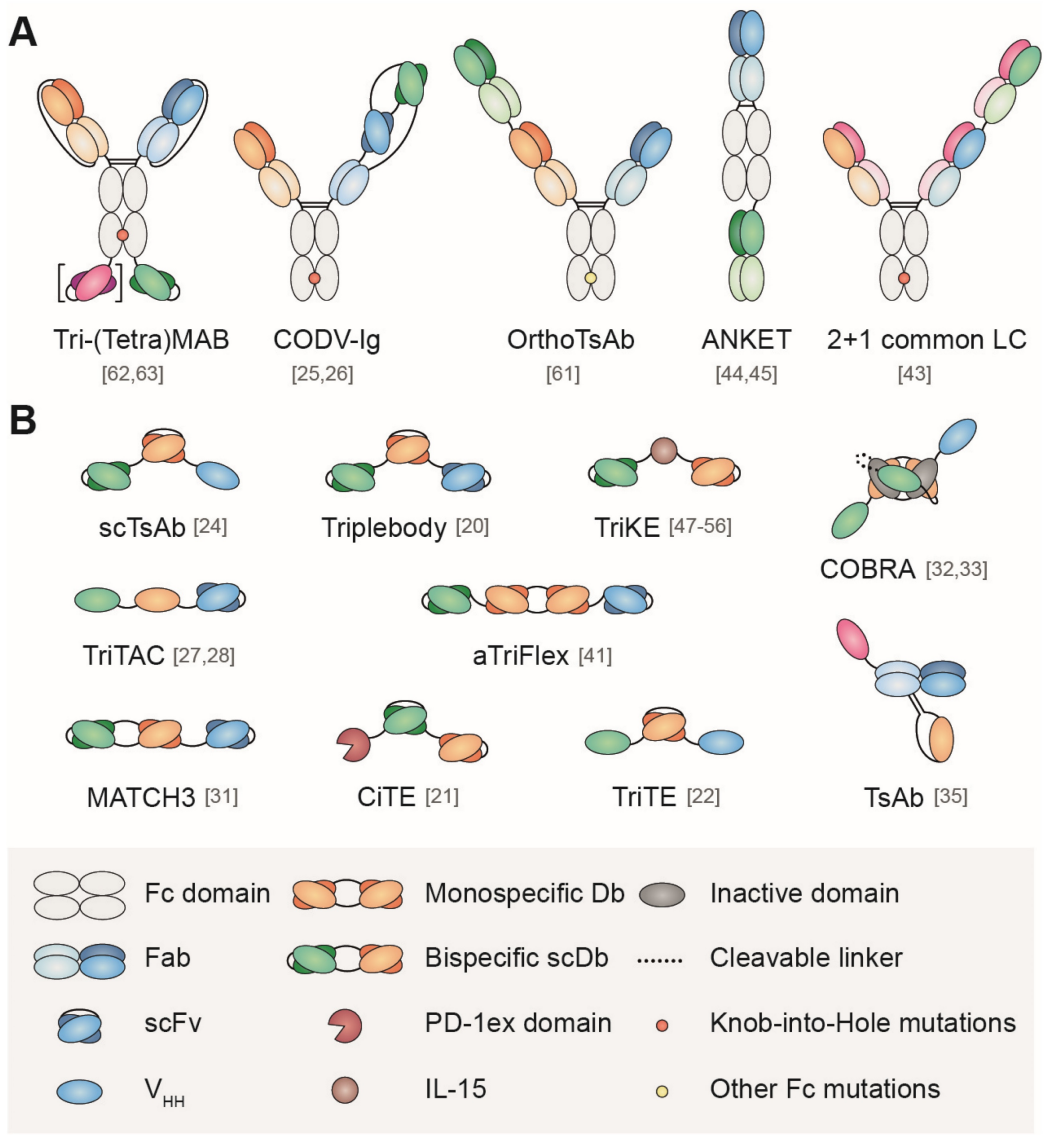
Schematic representation of some formats of multispecific antibodies with (A) or without (B) Fc domain. Numbers in brackets are the references to the original papers.

As previously described for BsAb 8,11, TsAb can be divided in IgG-like formats (with a Fc region) and non-IgG-like formats, (without a Fc region), according to their similarity with the conventional IgG structure (Figure 1). Non-IgG-like formats consists of antibody-derived building blocks fused via flexible linkers, the simplest arrangement being 'beads-on-string'. From higher to smaller molecular weight, these binding domains can be antigen-binding fragments (Fab), single-chain variable fragments (scFv) or single domain antibodies (sdAb). A comprehensive review on the building blocks used to tailor the design of multispecific Ab can be found in Wu *et al.* 12; here, we will only address some basic concepts.

Fab fragments (50 kDa), comprise the full-length light (L) chain (VL and CL domains) and VH and CH1 domains of the heavy (H) chain from a conventional IgG, connected by an interchain disulfide bond. On the other hand, scFv are moieties of 25-30 kDa, which contain only VH and VL domains joined by a flexible polypeptide linker, usually (GGGGS)_3_. Finally, sdAb (12-15 kDa) consist of a unique variable domain derived from heavy-chain only antibodies present in camelids (named VHH) or cartilaginous fish (named VNAR). Obviously, the non-human nature of sdAb fragments makes them potentially immunogenic, although humanization approaches have been developed to mitigate this problem. Coding sequences of variable domains can be obtained from IgG with known specificities or by selection *in vitro* of large Ab libraries using phage, bacterial or yeast display. Of note, scFv and sdAb are easily produced as soluble, properly folded and stable recombinant proteins in bacteria, yeast and mammalian systems, with suitable ranges of affinities 13,14.

Multimerization of these building blocks enables the modification of antibody size, shape, and valence to optimize half-life and tumor penetration. For instance, the tandem scFv or (scFv)_2_ BsAb format, consists of two scFv fused in a single polypeptide chain by a five-amino acid linker 14. Similarly, the assembly into a single Fc-less polypeptidic chain of three building blocks with different specificities allows the design of a variety of non-IgG-like TsAb formats, avoiding Fc-associated adverse effects in some contexts. In general, their smaller size in comparison with conventional IgG enhances tumor penetration, although at the cost of faster clearance 15.

On the other hand, IgG-like formats comprise molecules that partially or completely resemble natural antibodies, including a Fc region (functional or not). Designing Fc-based TsAb facilities dimerization, purification and promotes stability and extended half-life. Most of IgG-like formats consist of asymmetric chains, which include modifications in CH3 domains (such as the knob into hole -KIH- technology) in order to enforce the heterodimerization ot two different heavy chains. The correct pairing of light chains is a challenge that is addressed with different strategies. However, a percentage of non-functional molecules or undesirable homodimers can still cause impurities. BsAb in IgG format may gain a third specificity by appending an antibody fragment to the N- or C-terminal ends of heavy or light chains. However, the large molecular weight of IgG-like TsAb formats hampers tumor penetration, although guarantee long serum half-lifes.

## Mechanisms of action of trispecific antibodies for cancer treatment

The main application field of TsAb remains oncology, with at least one of the three specificities intended to bind T or NK cells, and at least one targeting a tumor-associated antigen (TAA) in most designs 16 (Table 1).

### Immune cell engagers (ICE)

Immune cell engagers are molecules able to redirect immune effector cells (regardless of their antigen specificity) against cancer cells with the aim of triggering efficient tumor cell killing, acting as a bridge between immune cells and target cells 68. Catumaxomab and blinatumomab were the first ICE approved for clinical use, both with a bispecific format and including an anti-CD3 moiety for T cell redirection. Substitution of anti-CD3 binding domain with an anti-CD16 one allows the recruitment of NK cells. In both cases, the addition of a third binding domain endows ICE with new and appealing properties, including increased specificity for tumors, or enhanced immune-cell activation.

#### T cells engagers (TCE)

##### Dual targeting of tumor cells by T cells

Beyond increasing the avidity and size to overcome fast clearance in small antibody fragment-based constructs, the incorporation of a second in-cis binding antibody moiety targeting a different TAA expressed on the same tumor cells offers additional advantages. For example, dual TAA-targeting may contribute to prevent tumor escape by antigen loss caused by selective pressure in comparison to conventional single TAA-targeting TCE and may help to overcome antigenic heterogeneity.

In a pioneering study**,** Schoonjans *et al.* described in 2000 a TsAb format, which they refer to as tribody, constituted by the fusion of two scFv against different TAA (BCL1 and hPLAP) to an anti-CD3 Fab, rendering an intermediate sized construct with a molecular weight around 100 kDa 17. Moreover, they showed concurrent binding to the three cognate antigens.

Subsequently, dual TAA-targeting TsAb have been designed for leukemic cell killing, as an evolution of the CD19/CD3 BiTE (Bispecific T-cell Engager) blinatumomab, a CD3-based tandem scFv. This BsAb has shown remarkable efficacy in patients with B-ALL, although the durability of the response is limited, and the relapse rate is ~50% after 12-18 months. Interestingly, around 30% of blinatumomab-relapsed cases are characterized by CD19 negative leukemic cells 69. To potentially overcome CD19 antigen escape, a second anti-CD20 scFv was added to a CD19/CD3 Fab-scFv BsAb, although TsAb efficacy *in vitro* was inferior to that of the BsAb 18. In another work, a CD19/CD22/CD3 TsAb was designed by fusing anti-CD19 scFv and anti-CD22 sdAb to a CD3 binding Fab 19. In this case, the TsAb was superior inducing T-cell specific cytotoxicity and cytokine production *in vitro* against CD19+ and/or CD22+ tumor cells and demonstrated significantly enhanced antitumor efficacy in a patient-derived xenograft (PDX) model of B-ALL, compared with the corresponding BsAb alone or in combination. Remarkably, leukemia relapse was observed in all animals, independently of the initial response, except in the group treated with the TsAb.

Leukemia cells with mixed B/myeloid origin are found in a small percentage and usually have poor prognosis. To deal with this condition, a CD33/CD3/CD19 TsAb, termed triplebody, was designed. Triplebodies consist of three scFv in tandem fused in a single polypeptide chain, with a molecular weight around 80-90 kDa. The two distal binding domains recognize different TAA, while the central domain recruits effector T cells. The CD33/CD3/CD19 triplebody showed efficient lysis of different ALL and AML cell lines, with enhanced selectivity for CD19+ CD33+ double-positive cells over CD19+ with comparable antigen density 20.

Fine-tuning affinity in TAA-binding domains can help to further increase tumor selectivity. Another TsAb designed for AML treatment, named CiTE (Checkpoint Inhibitory T cell-Engaging) Ab, combined T cell redirection to CD33 on AML cells with PD-L1 blockade on the same cells 21. In this study, a PD-L1/CD3/CD33 triplebody structurally similar to the referred above was compared to a trifunctional construct where the high-affinity anti-PD-L1 scFv had been substituted by the low-affinity PD-1 extracellular domain. In such a construct, PD-L1 binding was dependent on CD33 targeting, restricting checkpoint blockade to the surface of leukemic cells. Consequently, the CiTE only induced lysis of CD33+, PDL1+ cells *in vitro*, whereas PD-L1+ non-AML cells were spared. Moreover, the CiTE construct achieved complete AML eradication in a PDX model without immune-related adverse effects.

In another work, a TsAb for dual targeting in solid tumors, termed trispecific T-cell engager (TriTE), was designed. This construct consists of a CD3-specific scFv flanked by an anti-epidermal growth factor receptor (EGFR) sdAb and an anti-epithelial cell (EpCAM) sdAb 22. The TriTE enabled the specific cytolysis of EGFR- and/or EpCAM-expressing colorectal cancer cells. Bispecific bivalent targeting of double-positive HCT116 cells by TriTE improved *in vitro* potency up to 100-fold compared to single-positive cells and significantly prolonged survival *in vivo* compared to control BsAb. To avoid systemic toxicity, it has been proposed that a dual-TAA targeting BsAb should preferentially bind to malignant cells rather than normal cells if the affinity of the individual binding domains are sufficiently low as to require the presence of both target antigens (through the avidity effect) for efficient binding 70. According to this principle, the TriTE antibody was less efficient at killing single-positive target cells than the corresponding BsAb controls, leading to potentially enhanced tumor specificity.

Recently, Liu and coworkers reported a thorough strategy to determine the most suitable fusion site of two VHH (anti-HER2 and anti-VEGFR2) to an anti-CD3 Fab for optimal antigen recognition. The resulting TsAb induced not only a more durable growth inhibition of double-positive tumors compared with the combination of corresponding BsAb, but also overcame tumor heterogeneity and elicited tumor regression in mice coimplanted with HER2+ or VEGFR2+ tumors in each flank. Moreover, the TsAb-based treatment inhibited tumor growth in a PC3 tumor model resistant to the approved mAb trastuzumab (anti-HER2) and ramucirumab (anti-VEGFR2). These results further supported the rationale that TAA dual targeting can improve the therapeutic index of TsAb 35.

##### Targeting of two T cell costimulatory and/or checkpoint receptors

Antigen-independent stimulation of T cells with canonical bivalent anti-CD3 mAb was demonstrated decades ago. On the other hand, monovalent CD3 binding, as it occurs with TCE comprising a single CD3-targeting domain, can induce T cell activation only upon crosslinking to TAA on targets cells. However, this must be accompanied by co-stimulatory signals for optimal activation and T cell proliferation. Furthermore, activated T cells, in turn, express inhibitory checkpoint receptors to control the immune response. Hence, the design of trispecific TCE including agonists of co-stimulatory molecules or antagonists of checkpoint receptors, along with the anti-CD3 and anti-TAA moieties, constitutes an appealing strategy. Such molecules, comprising an agonist of the co-stimulatory receptor CD28 (TAA/CD3/CD28), were expressed as single-chain polypeptides (in scFv-sdAb-sdAb or scFv-scFv-sdAb formats) and characterized *in vitro* for the killing of ovarian 23 or colon 24 cancer cells, respectively.

However, it was the study by Wu *et al.* 25, building on previous work by this group on a TsAb anti-HIV 71, that brought this modality of TsAb into the spotlight 72. Using the Cross-Over Dual Variable (CODV) antibody format, different combinations of three binding domains (CD38/CD3/CD28) were mutated to assess the individual contributions of each specificity. To minimize the risk of cytokine release syndrome (CRS), the authors used the non-complement fixing IgG4 isotype, and introduced previously described mutations (F234A, L235A) into the Fc region to abrogate binding to Fc receptors. The presence of the anti-CD28 domain on the TsAb enhanced T cell activation and killing of different multiple myeloma (MM) cell lines *in vitro*, far above the potency of daratumumab (the anti-CD38 mAb approved for the treatment of MM). Similarly, the TsAb was superior in a humanized mouse model of MM, even at the lowest dose tested.

The use of CD28 agonists is associated to the risk of severe side effects due to cytokine release since the 2006 TeGenero clinical trial 73. Importantly, maximum MM cell killing *in vitro* was observed at CD38/CD3/CD28 TsAb concentrations far below serum levels well tolerated in non-human primates (NHP). The authors argue that monovalent CD28 binding by the TsAb is less prone to induce cytokine release than the bivalent anti-CD28 IgG used in the above-mentioned trial. This TsAb, coined SAR442257, is currently in a phase I clinical trial with MM patients which eventually will dilucidate if safety concerns are justified 74 (Table 2).

A second TsAb HER2/CD3/CD28, based on the same platform, has recently joined it in clinical trials. In a humanized breast cancer model, this TsAb mediated effective tumor regression, even in low HER2-expressing tumors, suggesting a potential benefit to breast cancer patients who are not candidates for current anti-HER2 therapies 26. Although CD8 T cells are widely accepted to be responsible for tumor cell elimination, an intriguing finding of this work was the main role of CD4 cells in the reported therapeutic effect.

##### TCE engineered for half-life extension

A third subtype of trispecific TCE comprises binding domains against a single TAA and a single T-cell activating receptor, along with a moiety for increased serum half-life. TsAb based on small Ab fragments, such as scFv and sdAb, usually exhibit molecular weights below the renal filtration cut-off, despite their multimeric nature. In fact, the 55-kDa BiTE blinatumomab has a serum half-life of only 2 hours and, therefore, continuous intravenous infusion is required 75.

The inclusion of albumin-binding sdAb is a widely used half-life extension strategy for small biotherapeutics. Albumin has a serum half-life of 3 weeks because of its size and FcRn-mediated recycling, properties shared with canonical IgG antibodies 76. Half-life extension of antibody fragments by fusion to an albumin-binding sdAb has been demonstrated in different species in preclinical studies 77. The great advantage of extended serum persistence is a more even drug concentration, less frequent dosing, and the potential to decrease doses without compromising therapeutical efficacy. Not surprisingly, a considerable proportion of TsAb currently in clinical trials have been half-life extended (HLE) using this strategy.

For example, the single-chain TriTAC (Trispecific T-cell Activating Construct) format, with a molecular weight of approximately 53 kDa, comprises a N-terminal humanized anti-TAA sdAb and a C-terminal humanized anti-CD3 scFv, separated by an anti-albumin sdAb which provides TriTAC with extended serum half-life. The serum levels of the anti-PSMA TriTAC HPN424 revealed a half-life of about 3.4 days in NHP 27 and up to 4.7 days for the anti-mesothelin TriTAC HPN536 28. As of November 2022, four TriTAC are in early phase clinical trials: the anti-BCMA (B cell maturation antigen) HPN217 for MM, the anti-DLL3 HPN328 for small cell lung cancer (SCLC), HPN424 for prostate cancer and HPN536 for mesothelin-positive solid tumors. In March 2022, the FDA granted Orphan Drug Designation to HPN328 for the treatment of patients with SCLC.

The MATCH3 (Multispecific Antibody-based Therapeutics by Cognate Heterodimerization) technology allows the assembly of three binding domains by fusion of a single-chain diabody to a scFv (scDb-scFv). NM21-1480 (PD-L1/CD137/HSA) combines in such a construct the ability to target CD137-mediated T cell costimulation to PD-L1 overexpressed in the tumor microenvironment (avoiding liver toxicity which hampers anti-CD137 IgG) and simultaneously blocking the PD-1/PD-L1 axis. Combination of HSA binding with a molecular weight around 80 kD endows these molecules with a suggested plasma half-life in humans of up to 2 weeks 31. NM21-1480 is being tested in Phase 1-2 clinical trial with non-small cell lung cancer (NSCLC) patients.

A similar strategy is exploited by the Humabody CB307, with three fully human sdAb targeting PSMA, CD137 and HSA (molecular weight <50 kDa), currently in phase I trial for the treatment of advanced PSMA+ tumors. CB307 functions as a CD137 agonist selectively in the presence of PSMA+ cancer cells, thereby enabling tumor-specific T cell activation and decreasing the risk of systemic side effects. Based on the same technology, CB213 is a tetravalent trispecific Humabody engaging simultaneously PD1 and LAG3 on T cells and HSA. CB213 showed superior activity compared to an anti-PD1 mAb to induce *ex vivo* proliferation of T cells from NSCLC patients and to suppress tumor growth *in vivo*. In addition, NHP pharmacokinetics suggested a potential weekly clinical administration, which is a favorable regimen considering the small size of these molecules 30.

However, non-covalent HSA binding by sdAb is susceptible to competition and displacement by endogenous ligands and other albumin binding drugs. In contrast, the genetic fusion of drugs to recombinant HSA offers the opportunity to control half-life by the inclusion of albumin sequences with different FcRn binding affinities 78. Recently, a new class of tumor-specific CD137 agonists was generated, named light T cell costimulatory (LiTCo) antibodies, composed of an anti-EGFR sdAb and a CD137-specific scFv. Genetic fusion to a human albumin sequence engineered for high affinity FcRn binding (LiTCo-Albu) demonstrated a prolonged circulatory half-life of 30 hours and *in vivo* tumor inhibition 29.

#### NK cells engagers (NKCE)

Multispecific molecules targeting one (or two) activating NK cell receptors and a TAA constitute another type of ICE, developed to enhance tumor cell recognition and killing by NK cells 79. Among activating NK cell receptors, a hierarchy of CD16 > NKp46 > NKG2D has been established on the basis of their capability to trigger resting NK cells 80. Most of the constructs that address CD16 (FcγRIIIa) on NK cells contain a CD16-specific scFv moiety instead of an IgG Fc domain, the natural ligand for CD16, to avoid side effects associated with the Fc domain 81. Moreover, previous works have demonstrated that directly engaging CD16 on NK cells with a scFv can be more potent than CD16 engagement of Fc domains 82 and even have better safety profiles 74.

##### Dual targeting of tumor cells by NK cells

As in TCE, concurrent binding to two different TAA has also been implemented in NKCE antibodies to increase their therapeutic window. Addition of an extra anti-CD19 scFv to a BsAb CD19/CD16 rendered a construct with a molecular weight around 90 kDa termed single-chain Fv triplebody 83. Incorporation of the second tumor-binding scFv led to increased avidity for CD19+ leukemia cells, promoted NK-mediated antibody-dependent cellular cytotoxicity (ADCC), and improved serum half-life. Dual targeting of AML cells was then been pursued with a TsAb CD33/CD16/CD123, which induced significantly stronger NK lysis of primary leukemic cells than the trivalent BsAb CD123/CD16/CD123 (38). Humanized versions of the three original murine scFv were used to produce the clinical candidate CD33/CD16/CD123 triplebody SPM-2 39. Additional triplebodies have been reported targeting simultaneously CD33 or HLA-DR and CD19, and recruiting NK cells via an anti-CD16 moiety 37,84 or using a NKG2D ligand 36. A CD16/CD22/CD19 Ts Ab also based on a tandem scFv format was generated for the NK-mediated elimination of different types of leukemic cells 40.

Another CD16-directed trispecific, but tetravalent antibody format, termed aTriFlex, has been generated for the redirection of NK cell cytotoxicity to two multiple myeloma antigens. This format consists of an anti-CD16 monospecific diabody with two scFv in both ends, one anti-BCMA and another anti-CD200. Dual-targeting of multiple myeloma cells resulted in enhanced selectivity and 17-fold increase in potency 41.

IgG-like TsAb in this section include a sdAb-based SEEDbody, generated using the Strand-Exchanged Engineered Domains (SEED) heterodimerization technology, by replacement of the VH and VL regions of a conventional antibody by two different sdAb domains and grafting a third sdAb onto the hinge region of the second heavy chain 42. As a proof of principle, an EGFR/HER2/NKG2D TsAb, which simultaneously bound each antigen, was obtained. Another TsAb was generated with the common light chain technology for the simultaneous targeting of EGFR, CD16 and PD-L1. This TsAb was based on a BsAb EGFR/CD16 IgG with an anti-PD-L1 arm N-terminally fused to the anti-CD16 Fab. The Fc-based approach was chosen to endow the molecule with prolonged half-life, mediated by the elevated molecular size (around 200 kDa) and FcRn-mediated recycling. From the mechanistical point of view, it should be noted that the PD-1/PD-L1 axis is an immune checkpoint for not only T cells but also for NK cells, and EGF signaling can induce PD-L1 upregulation. Simultaneous targeting of EGFR and PD-L1 on the same tumor cell increased TsAb avidity and tumor specificity, along with checkpoint inhibition, which translated into a more potent ADCC effect *in vitro* compared to the parental BsAb 43.

##### Dual Targeting of NK cell activation receptors

Full activation of NK cells to induce anti-tumor immunity has been shown to require the co-engagement of different cell-surface receptors. Based on this consideration, a series of molecules which bind a TAA and trigger two NK cell activation receptors have been designed.

The Antibody-based NK cell Engager Technology (ANKET) platform has generated a range of promising NKCE targeting different tumor antigens. In a pioneering work, Gauthier *et al.* reported the characterization of a trifunctional NKCE consisting of two Fab antibody fragments targeting NKp46 and a TAA (CD19, CD20 or EGFR), separated by an Fc domain to promote ADCC via CD16 44. Thereby, this format cannot be strictly considered as trispecific since it lacks an anti-CD16 moiety. These trifunctional molecules were effective against several tumor types *in vitro*, with no off-target cytotoxicity. In Raji B lymphoma models, mice treated with Fc-silenced NKCE or trifunctional NKCE controlled disease better than the group treated with the mAb rituximab, but the trifunctional construct was significantly more potent and outperformed a mixture of reagents activating NKp46 and CD16 separately. In a related work, trifunctional NKCE were designed targeting CD19 or CD20 and triggering either NKp46 or NKp30, aimed to the treatment of pediatric B-ALL 45. *In vitro* experiments demonstrated efficient NK cell-mediated killing by all NKCE of leukemia cell lines and primary blasts, including the NK cell-resistant MHH-CALL-4. Moreover, a clinical study using an anti-NKp46-based ANKET targeting CD123 (IPH6101/SAR443579) is ongoing in patients with AML (NCT05086315). Another trifunctional ANKET (IPH6401/SAR'514), directed against BCMA, has recently been selected for clinical development.

A different platform for the development of multifunctional NKCE has been named TriNKET (Trispecific NKCE Therapies). DF1001 is one of several agents belonging to this platform, which targets HER2 and coengages CD16 and NKG2D 85. The safety and efficacy of this HER2-targeting TriNKET are being investigated in a Phase 1/2 trial in patients with various advanced-stage HER2+ solid tumors, alone or in combination with immune checkpoint inhibitors.

##### IL-15-based trifunctional NK cells engagers

TriKEs (Trispecific Killer cell Engagers) are not strictly speaking TsAb, since they bind a TAA with one arm and CD16 with the other, while their third moiety is IL-15, which activates the recruited NK cells via interaction with the corresponding receptor. Nevertheless, since they are antibody-based, trifunctional molecules, they will be considered here, as proposed by Elsiathy *et al.* 16.

The first IL-15-based TriKE reported in the literature was an evolution of a previously characterized BiKE (Bispecific Killer cell Engager) based on a tandem scFv CD33/CD16 47. When compared with the BiKE, the TriKE induced superior NK cell cytotoxicity, degranulation, and cytokine production against the CD33+ AML cell line HL-60 and increased NK survival and proliferation *in vitro*. In a HL-60 mouse tumor model, the TriKE also showed enhanced antitumor activity relative to BiKE. Interestingly, TriKE-treated animals had substantial numbers of circulating human NK cells three weeks after, when they were hardly detectable in untreated or BiKE-treated animals. A phase 1 clinical trial (NCT03214666) with this TriKE, named GTB-3550, demonstrated absence of severe side effects, expansion of NK cells and clinical effect in patients with AML, with significant reduction in the numbers of leukemic blasts in the bone marrow 48. However, it was terminated due to development of a second-generation TriKE. Other three first generation TriKE were generated against EpCAM 49, with activity in breast, prostate, ovarian and head and neck carcinomas; CD133, for the elimination of cancer stem cells 50; and CD19, for the treatment of CLL 51.

Second generation TriKE were based on a humanized anti-CD16 sdAb, since the camelid CD16-engaging moiety provided further potency than the corresponding scFv 56. A CD33-targeting second-generation TriKE induced more potent NK cell proliferation than its first-generation counterpart and showed enhanced tumor control in preclinical mouse models. This TriKE, coined GTB-3650, is the first clinical candidate being developed using this technology. Similarly, other second generation TriKE prototypes contain moieties anti B7-H3 for ovarian and lung carcinoma 52; anti-CLEC12A, for AML 54; and anti-HER2, for breast and ovarian carcinoma 53.

Very recently, it has been reported a second-generation TriKE with a novel mechanism of action, directed against the stroma-associated antigen TEM8 (Tumor Endothelial Marker 8) 55. TEM8 is expressed on tumor and tumor stromal cells (endothelial cells, fibroblasts and pericytes) in the tumor microenvironment (TME) of different types of cancers. This TriKE selectively promoted NK cell degranulation, cytokine secretion and NK cell-mediated inhibition of tumor growth and tumor angiogenesis in A549 lung cancer xenograft models, along with increased numbers of tumor-infiltrating NK cells.

### Targeting multiple receptors in tumor cells or immune effector cells

In this section, we will review TsAb which do not bridge tumor and effector cells, as they recognize in-cis antigens expressed on the surface of the same cell.

#### Multitargeting of tumor cells

Small molecule EGFR tyrosine kinase inhibitors are used for the treatment of NSCLC driven by EGFR mutations, but resistance frequently appears due to different mechanisms such as cMET amplification. The BsAb amivantamab, which recognizes EGFR and cMet and blocks their respective signalling pathways, was approved in 2021 for the treatment of NSCLC with EGFR exon 20 insertion mutations. The incorporation of a third binding domain to an in-cis targeting TsAb could potentially increase its efficacy. Indeed, a HER2/EGFR/cMet TsAb has been obtained using Fab interface designs to induce correct chain pairing, denoted OrthoTsAb. TsAb were designed with varied geometries, and overall, every construct demonstrated strong trispecific binding although their therapeutic potential was not addressed 61. However, in the configuration with the anti-cMet Fab at the C-terminus of the TsAb, binding to this antigen was considerably impaired, suggesting steric inhibition of epitope recognition. Interestingly, the positioning effect on the binding affinity of the anti-EGFR V_HH_ fused in the C-terminal end of TsAb has been previously described by different groups 22,27. Modification of linker length and/or composition could be a strategy to improve the kinetic on-rates for binding moieties in unfavorable positions.

Another example of multiple tumor targeting is the IgG-like TriMAb directed against EGFR, IGF1R and cMet or HER3 62. The TsAb inhibited ligand-dependent receptor phosphorylation similarly to the parental antibodies. However, in a proliferation assay comparing TriMAb activity with individual antibodies or combinations, a superior growth inhibitory effect of the EGFR/IGF1R/HER3 TriMAb was observed. In addition, CD16 binding to the Fc domain of TriMAb was not impaired by scFv fusions at the C-terminus of the heavy chains.

A different approach is under the design of GB263T, an IgG-like TsAb recognizing EGFR and two different cMET epitopes, with enhanced ADCC function. *In vitro*, the TsAb blocked phosphorylation of both receptors and inhibited the proliferation of NSLC cells with different EGFR mutations. Significant *in vivo* anti-tumoral efficacy of GB263T was shown in several tumor models of EGFR exon 20 insertion, EGFR C797S mutation, cMET amplification, and cMET exon 14 skipping mutation. Currently, GB263T is under clinical testing in a phase 1 study (NCT05332574) 64.

On the other hand, ephrin (Eph) receptors constitute another family of tyrosine kinase receptors less explored as therapeutic targets, with EphA2, EphA4, and EphB4 found overexpressed in different types of carcinoma 65. In the design of a TsAb targeting these three receptors, the anti-EphB4 and anti-EphA4 variable domains in a diabody configuration were linked to the C-terminal end of the heavy chains of a full-length anti-EphA2 IgG, rendering a bulky molecule around 250 kDa. Concurrent binding of the TsAb to the three antigens was demonstrated using BIAcore, although it did not translate into enhanced antitumor activity over parental antibodies.

An interesting approach could be the use of TsAb-based immunoconjugates for tumor delivery of drugs, toxins or radioisotopes, potentially increasing selectivity and therefore their therapeutic window.

#### Multitargeting of effector cells

Immunomodulatory mAb include immune checkpoint inhibitors and agonists of costimulatory receptors. Given the nonredundant signaling of the respective pathways, it is conceivable that combinations of agonist and antagonist antibodies could synergize to obtain higher response rates 86,87. And doing so with a single molecule may offer additional benefits, although concerns about systemic unspecific T cell activation could rise since these constructs lack an anti-TAA moiety to redirect them. In the same work by Wu *et al.* the generation of a TsAb combining antagonists of PD-1 and CTLA-4 with a CD137 agonist was reported, although it was not functionally characterized 61.

### Other mechanisms of action

Combinations of three different binding moieties in a single molecule offer a wide variety of new therapeutic options. A good example is the TsAb HER2/FAP/mPEG (scFv/scFv/Fab format) for conversion of mPEG (methoxypolyethylene glycol)-coated liposomes containing doxorubicin to immunoliposomes directed towards HER2+ breast cancer cells and tumor-associated fibroblasts (TAF) expressing FAP (fibroblast activation protein) 67. Notably, the TsAb-decorated liposomes inhibited the growth of tumors containing TAFs (but not TAF-free tumors) more efficiently than liposomes modified with the control BsAb HER2/mPEG or FAP/mPEG.

## Challenges and Perspectives

### Beyond three different specificities

If antibody engineering allows the assembly in a single functional molecule of three different binding domains, adding a fourth one would not be a cumbersome task. Indeed, tetraspecific Ab (TtsAb) are not a future perspective any longer, but a reality. So real that some of them have reached clinical testing.

One of the most interesting platforms for this purpose is named CrossMAb. This technology is based on the crossover of VH and VL or CH1 and CL domains in one Fab arm of a Bs IgG to promote the correct assembling of light chains, along with the KIH technology to enforce the heterodimerization of heavy chains 88. The 4-in-one CrossMab targeting EGFR, HER2, HER3 and VEGF 89 showed superior antitumor activity in different cancer models *in vivo* relative to combinations of BsAb, and they did so at lower antibody concentrations enabled by higher-avidity binding of tumor cells.

Another format amenable to TtsAb is coined MATCH4, which can be applied to produce homogeneous and stable antibody-derived molecules 34. This concept is based on a core of 2 split variable domain pairs, with each chain containing either 2 VL domains or 2 VH domains in tandem, thereby driving heterodimerization of both chains. Additional scFv appended to the N-terminal end of both chains (or the N-terminus of one chain and the C-terminus of another) provide the two extra specificities. In a proof-of-concept study, tetraspecific heterodimers were generated in this combination with specificities for human TNFα, CD3, IL-5R and IL-23R. Antigen affinities of the binding domains in the MATCH formats were similar to those of the corresponding scFvs and single chain diabodies (scDbs), and the tetraspecific molecules were able to bind all target antigens simultaneously, irrespective of the order in which they encounter antigen.

The Tetramab concept is based on the combination of single-chain Fab and Fv fragments in an IgG antibody format enhanced by KIH technology 63. A TsAb targeting HER3, cMet, HER1 and IGF1R was designed with the potential to avoid compensation mechanisms between different RTK signaling pathways. This construct was able to bind the cognate antigens with affinities in the nM range, comparable to the parental monospecific antibodies, and showed inhibition of RTK activation in pancreatic, mammary and lung tumor cells in presence of the corresponding growth factors. Importantly, the TetraMab showed improved tumor growth inhibition over individual monospecific or BsAb in co-cultures of different NSCLC cell lines.

Tetraspecific NKCEs also come in different formats. The DuoBody (DB)-VHH incorporates VHH with different specificities attached to the heavy chain C-terminal ends of a bispecific, Fc-silent IgG 58. One or two VHH against EGFR, IL6R or NKG2D were appended to a HER2/cMET BsAb. Tri-and tetraspecific DB-VHH demonstrated binding to their cognate antigens and the NKG2D constructs showed promising results of NK cell-mediated cytotoxicity *in vitro*, although not significant differences were observed among them.

An evolution of TriKE format is TetraKE, which has been described to simultaneously engage EpCAM and CD133 for targeting carcinoma cells and cancer stem cells, along with a CD16-engaging moiety and IL-15 for NK cell expansion 59. However, NK cytotoxic activity against EpCAM+, CD133+ Caco2 cells promoted by the TetraKE was similar to that of the EpCAM/CD16 BiKE.

Furthermore, a new type of multifunctional molecule based on the ANKET technology (named ANKET4) incorporates an IL-2 variant, binding IL-2 receptor β and γ chains, to NKp46 and CD16 co-engagement and targeting of a single TAA. ANKET4 induced NK cell proliferation and accumulation at the TME and had a higher anti-tumor efficacy than approved therapeutic antibodies targeting the same TAA. In NHP, a CD20-directed ANKET4 resulted in sustained CD20+ B-cell depletion with minimal systemic cytokine release and no clinical sign of toxicity 46.

On the other hand, the TriTECM platform enables the fusion of trispecific TCE to an anti-IL6R scFv antibody in order to mitigate the CRS mainly promoted by IL6. It is well known that on-target T cell activation is associated with release of cytokines that can potentially result in CRS, being this one of the major safety concerns intrinsic to the mode of action of TCE. Several EGFR/PD-L1/CD3/IL6R TriTECM candidates were generated by fusing one or two anti-IL6R scFv to the TsAb and ensuring the heterodimerization using KiH technology. In both cases, TriTECM antibodies inhibited simultaneously and dose-dependently EGFR and IL6R signaling pathways and triggered efficiently T cell activation and cytotoxicity against EGFR+ cells. In addition, TriTECM-mediated antitumor T cell activity was attenuated in comparison with EGFR/PD-L1/CD3 TsAb variants, indicating TriTECM potential as TCE with cytokine release modulator activity 90.

A search in clinicaltrials.gov as of November 2022 renders at least three tetraspecific TCE: GNC-038 (CD3/41BB/PD-L1/CD19), being tested in phase 1 trials with patients with NHL (NCT04606433) and DLBCL (NCT05192486); GNC-035 (CD3/41BB/PD-L1/ROR1) in patients with breast cancer; and GNC-039 (CD3/41BB/PD-L1/EGFRvIII) in glioma (NCT05160545).

### Nonimmunoglobulin-trispecific binding proteins

An alternative to Ab-based constructs is the use of non-immunoglobulin scaffolds as building blocks 91. Among the most advanced scaffold protein platforms are anticalins, affibodies and DARPins. DARPins are based on naturally occurring ankyrin repeat domains, which constitute a scaffold amenable to the generation of multispecific proteins 92. Two trispecific DARPins are in clinical trials: MP0310 (CD137/FAP/HSA) and MP0317 (CD40/FAP/HSA). The first is intended to localize CD137 agonistic activity to FAP+ tumor-associated macrophages (TAM) to preferentially activate T cells in the TME. The second aims to selectively activate CD40 bearing B-cells, dendritic cells, and macrophages in the presence of FAP. In both cases, the objective is to avoid toxicity associated with systemic activation. Interestingly, suppressive TAM were repolarized by MP0317 to an anti-tumoral phenotype, restoring T cell responses 93,94.

### Conditionally active trispecific T-cell engagers

Another interesting approach to widen the therapeutic window of TsAb is the use of conditionally active TCE, administered as inactive prodrugs, and designed for prolonged release (to avoid CRS) or tumor-restricted activation (to avoid on target-off tumor side effects) 95. One example of the first option is the TriTAC-XR platform, which become slowly activated in systemic circulation, reducing the maximum drug concentration and prolonging dosing intervals, similar to subcutaneous dosing 96. The prodrug was obtained by the incorporation of a peptide mask which binds to the anti-CD3 moiety and a protease-cleavable linker to the N-terminus of a TriTAC. Active TCE is released after cleavage of the linker by systemic proteases. In NHP, TriTAC-XR showed significantly reduced cytokine production while maintaining comparable pharmacodynamic properties as a non-masked TCE.

Other strategies include proteolytic cleavage sites in the TCE design to take advantage of the enhanced protease activity in the TME, as the ProTriTAC platform. An alternative to the masking peptide has been named COBRA (COnditional Bispecific Redirected Activation) where anti-CD3 VH and VL domains are forced to pair with inactive VL and VH domains in a diabody-like structure. Anti-EGFR and anti-HSA sdAb were added to the N- and C-terminal ends in a two-chain or single chain configuration 32. MMP-9 release of the inactive domains allowed assembly of active anti-CD3 VH and VL domains on the surface of tumor cells. Complete regressions in colorectal or head and neck squamous cell carcinoma xenograft models were observed after treatment with the clinical COBRA candidate TAK-186, but not with the construct bearing non-cleavable linkers. Furthermore, TAK-186-mediated antitumoral effect was found to be dependent on the EGFR expression levels in tumors 33. Currently, TAK-186 is the first and only trispecific conditionally active TCE in clinical trials in patients with EGFR+ solid tumors (NCT04844073).

Another EGFR/CD3/HSA trispecific construct named tumor-activated T cell engager (TRACTr) 97 contains two protease cleavable masks that inhibit both EGFR binding on target cells and CD3 engagement on T cells. Both cleavable JANX008 TRACTr and the non-masked construct induced complete eradication of HCT116 colorectal (CRC) tumors in mice, although the TRACTr exhibited enhanced safety and pharmacokinetic properties relative to the EGFR-TCE in NHP.

Finally, TwoGATE (Two component Guided Antibody Tumor Engager) platform consists of two HLE BsAb each targeting a different TAA and forming an active anti-CD3 moiety only when both bind to a cancer cell. Each of the CD3-targeting split paratopes are associated with an inactivating domain cleaved by tumor-specific proteases in the TME.

In all approaches described above, the HLE domain (usually, an anti-HSA antibody fragment) is lost upon proteolytic activation, reducing the risk of systemic side effects if the active form of the TCE is released from the tumor.

## Concluding remarks

The development of complex therapeutics such as TsAb and TtsAb offers important challenges in terms of manufacturability. Some constructs may suffer from compromised stability, resulting in unfolding and aggregation. In addition, production of multichain multispecifics may be hampered by non-desired by-products arising due to incorrect assembly. Good manufacturing practice-compliant processes should be optimized to solve these problems, although in some cases production yields for TsAb were found to be better than for BsAb 74.

Many of the studies commented in this review are only proof-of-concept, focusing mainly on the design, expression, and preliminary functional characterization of TsAb, but a portion of them offer compelling *in vivo* data, and overall, they reveal the growing interest in the TsAb field that has blossomed in recent years. Indeed, multispecific antibodies are expanding the toolbox of antitumor reagents given their improved ability to target the multifactorial nature of cancer.

Specially interesting will be the demonstration of the TsAb therapeutic effect in non-hematological cancers compared to leukemias or lymphomas, currently a challenge for more advanced clinical trial-stage strategies such as BsAb and CAR-T cells, hampered by the lack of suitable single target antigens, intratumoral heterogeneity and the hostile tumor microenvironment promoting exclusion and/or exhaustion of recruited effector cells. It can be expected that TsAb, in their variety of flavors, will be better equipped to deal with these hurdles.

Ongoing early-stage clinical trials, most of them in patients with solid tumors, will provide relevant information in the next future on the added value of TsAb compared to the plethora of immunotherapies in development.

## Figures and Tables

**Table 1:** Tri- and tetraspecific antibodies in preclinical development

Name	Format	Specificity	Model	Ref
**T cell-redirecting**				
TsAb	F(ab)3	CD2/CD3/CD37	LNH	Tutt *et al.* 9
Tribody	Fab-(scFv)2	BCL1/hPLAP/CD3	Mouse lymphoma	Schoonjans *et al.* 17
A-2019	scFv-Fab-scFv	CD19/CD3/CD20	B-ALL	Wang *et al.* 18
TsAb	scFv-Fab-Nb	CD19/CD22/CD3	B-ALL	Zhao *et al.* 19
triplebody	(scFv)3	CD33/CD3/CD19	MLL	Roskopf *et al.* 20
CiTE	PD-1ex-scFv-scFv	PD-L1/CD3/CD33	AML	Herrmann *et al.* 21
TsAb (TriTE)	VHH-scFv-VHH	EpCAM/CD3/EGFR	CRC	Tapia-Galisteo *et al.* 22
TsAb	F(ab)3	TA/CD3/CD28	Melanoma	Jung *et al.* 10
TR3H (scTsAb)	scFv-VH-VH	OCA/CD3/CD28	Ovarian cancer	Liu *et al.* 23
scTsAb	scFv-scFv-VH	CEA/CD3/CD28	CRC	Wang *et al.* 24
CD38 TsAb	Fab-Fc-CODV-Fab	CD38/CD3/CD28	MM, NHL	Wu *et al.* 25
HER2 TsAb	Fab-Fc-CODV-Fab	HER2/CD3/CD28	HER2+ solid tumors	Seung, *et al.* 26
TriTAC	VHH-VHH-scFV	PSMA/HSA/CD3	Prostate cancer	Austin *et al.* 27
TriTAC	VHH-VHH-scFV	MSLN/HSA/CD3	MSLN+ tumors	Molloy *et al.* 28
LiTCo-Albu	VHH-scFv-Albumin	EGFR/CD137/HSA	CRC	Hangiu *et al.* 29
CB213 (humabody)	(Human VH)4	LAG3/LAG3/PD1/HSA	CRC	Edwards *et al.* 30
MATCH3 (TsAb)	Db-scFc	CD137/PDL-1/HSA	NSCLC	Warmuth *et al.* 31
COBRA	VHH-scFc-VHH	EGFR/CD3/HSA	CRC	Panchal *et al.* 32; Dettling *et al.* 33
MATCH4 (TtsAb)	scFv-Db-scFv	IL-23R/IL-5R/TNFa/CD3	N/A	Egan *et al.* 34
TsAb	VHH-Fab-VHH	HER/VEGFR2/CD3	Breast, prostate cancer	Liu *et al.* 35
**NK cell-redirecting**				
triplebody	ULBP2-scFv-scFv	NKG2D/CD19/CD33	MLL	Vyas *et al.* 36
triplebody	(scFv)3	CD33/CD16/CD19	MLL	Schubert *et al.* 37
triplebody (sctb)	(scFv)3	CD123/CD16/CD33	AML	Kugler *et al.* 38
triplebody SPM-2	(scFv)3	CD33/CD16/CD123	AML	Braciak *et al.* 39
TriKE	(scFv)3	CD16/CD22/CD19	B-ALL, B-CLL, AML	Gleason *et al.* 40
ATriFlex	scFv-diabody-scFv	BCMA/CD200/CD16A	MM	Gantke *et al.* 41
SEEDbody	IgG-like VHH-based	EGFR/HER2/NKG2D	Breast cancer	Pekar *et al.* 42
TsAb	Bs IgG-Fab	EGFR/CD16a/PD-L1	Epidermoid carcinoma	Bogen *et al.* 43
ANKET	Fab-Fc-Fab	NKp46/CD16/CD19-CD20-EGFR	LNH	Gauthier *et al.* 44
ANKET	Fab/Fc/Fab	NKp46-NKp30/CD16/ CD19-CD20	B-ALL	Colomar *et al.* 45
ANKET4	Fab/Fc/Fab	NKp46/CD16/CD20/IL-2v	B-ALL	Demaria *et al.* 46
TriKE (GTB-3550)	scFv/IL-15/scFv	CD16/IL-15/CD33	AML	Vallera *et al.* 47; Felices *et al.* 48
TriKE	scFv/IL-15/scFv	CD16/IL-15/EpCAM	Various carcinomas	Schmohl *et al.* 49
TriKE	scFv/IL-15/scFv	CD16/IL-15/CD133	Cancer stem cells	Schmohl *et al.* 50
TriKE	scFv/IL-15/scFv	CD16/IL-15/CD19	B-CLL	Felices *et al.* 51
TriKE	VHH/IL-15/scFv	CD16/IL-15/B7H3	Ovarian cancer	Vallera *et al.* 52
TriKE	VHH/IL-15/scFV	CD16/IL-15/HER2	Ovarian cancer	Vallera *et al.* 53
TriKE	VHH/IL-15/scFV	CD16/IL-15/CLEC12A	AML	Arvindam *et al.* 54
TriKE	VHH/IL-15/scFV	CD16/IL-15/STEM8	NSCLC	Kaminski *et al.* 55
TriKE (GTB-3650)	VHH/IL-15/scFV	CD16/IL-15/CD33	AML, MDS	Felices *et al.* 56
HLE-nano-BiKE	(VHH)3	CD38/CD16/HSA	MM	Hambach *et al.* 57
DuoBody (DB)-VHH (TtsAb)	bs IgG-(VHH)2	HER2/cMET/EGFR-IL6R-NKG2D	Breast cancer	Yanakieva *et al.* 58
TetraKE (TtsAb)	scFv/IL-15/scFv/scFv	CD16/IL-5 /EpCAM/ CD133	CRC	Schmohl *et al.* 59
**Oher MOA**				
TAC	F(ab)3	EGFR/HER2/CD64	Breast cancer	Somasundaram *et al.* 60
OrthoTsAb	Fab-Fab-Fab; bs IgG-Fab	PD-1/CTLA-4 /CD137;HER-2/EGFR/cMet	N/A	Wu *et al.* 61
TriMAb	Bs IgG-scFv	EGFR/IGF1R/cMet-HER3	Pancreas cancer	Castoldi *et al.* 62
TetraMab	Bs IgG-(scFv)2	EGFR/IGF1R/cMet/HER3	NSCLC	Castoldi *et al.* 63
GB263T	enhanced Fc/NA	EGFR/cMET/cMET*	NSCLC	Du *et al.* 64
TsAb	IgG-(scDb)2	EphA2/EphB4/EphA4	Prostate cancer	Dimasi *et al.* 65
mAb^2^, Fab-exchanged TsAb	Fab-Fcab-Fab	EGFR/VEGF/Her2	Epidermoid carcinoma	Natale *et al.* 66
TsAb	scFv/scFv/Fab	HER2/FAP/mPEG	Breast cancer	Chen *et al.* 67

*Binds two different cMet epitopes.

**Table 2:** Tri-and tetraspecific antibodies in clinical trials as of Nov 2022

Drug name	Specificity	Indication	Clinical trial	Phase	Sponsor
SAR442257 (CODV-Fab)	CD38/CD3/CD28	MM, NHL	NCT04401020	1	Sanofi
SAR443216 (CODV-Fab)	HER2/CD3/CD28	HER2+ solid tumors	NCT05013554	1	Sanofi
SAR443579 (ANKET)	CD123/CD16/NKp46	AML, MDS	NCT05086315	1/2	Sanofi
CB307 (Humabody)	PSMA/CD137/HSA	PSMA+ tumors	NCT04839991	1	Crescendo Biologics
HPN217 (TriTAC)	BCMA/HSA/CD3	MM	NCT04184050	1	Harpoon Therapeutics
HPN328 (TriTAC)	DLL3/HSA/CD3	SCLC	NCT04471727	1/2	Harpoon Therapeutics
HPN424 (TriTAC)	PSMA/HSA/CD3	Prostate cancer	NCT03577028	1/2	Harpoon Therapeutics
HPN536 (TriTAC)	MSLN/HSA/CD3	MSLN+ tumors	NCT03872206	1/2	Harpoon Therapeutics
MP0317 (DARPin)	CD40/FAP/HSA	Advanced Solid Tumors	NCT05098405	1	Molecular Partners
MP0310 (DARPin)	CD137/FAP/HSA	Advanced Solid Tumors	NCT04049903	1	Molecular Partners
GTB-3550 (TriKE)	CD16/IL-15/CD33	AML	NCT03214666*	1/2	GT Biopharma
DF1001 (TriNKET)	HER2/CD16/NKG2D	HER2+ solid tumors	NCT04143711	1/2	Dragonfly Therapeutics
GB263T	EGFR/cMET/cMET*	NSCLC	NCT05332574	1/2	Genor Biopharma
NM21-1480 (scMATCH3)	PDL-1/CD137/HSA	NSCLC	NCT04442126	1/2	Numab Therapeutics
GNC-035	CD3/CD137/PD-L1/ROR1	Breast cancer	NCT05160545	1	Sichuan Baili/Systimmune
GNC-038	CD3/CD137/PD-L1/CD19	NHL	NCT04606433	1	Sichuan Baili/Systimmune
GNC-039	CD3/CD137/PD-L1/EGFRvIII	Glioma	NCT04794972	1	Sichuan Baili/Systimmune
TAK-186	EGFR/CD3/HSA	CCR, NSCLC, SCCHN	NCT04844073	1/2	Takeda

*Terminated, due to development of the second-generation TriKE GTB-3650.

## Abbreviations

B-ALL: B acute lymphoid leukemia
AML: acute myeloid leukemia
ANKET: antibody-based NK cell engager therapeutics
BCMA: B-cell maturation antigen
BiTE: bispecific T-cell engager
BsAb: bispecific antibody
B-CLL: B chronic lymphoid leukemia
CiTE: checkpoint inhibitory T cell-engaging
CODV: cross-over dual variable format
CRC: colorectal cancer
CRS: cytokine release syndrome
Db: diabody
EGFR: epidermal growth factor receptor
EpCAM: epithelial cell adhesion molecule
Fab: antigen-binding fragment
FAP: fibroblast activation protein
Fcab: antigen-binding Fc fragment
HAS: human serum albumin
mAb: monoclonal antibody
MATCH: multispecific antibody-based therapeutics by cognate heterodimerization
MDS: myelodysplastic syndrome
MLL: mixed lineage leukemia
mPEG: methoxypolyethylene glicol
MM: multiple myeloma
MSLN: mesothelin
NHL: non-Hodgkin lymphoma
NSCLC: non-small cell lung cancer
OCA: ovarian cancer antigen
PD-1ex: PD-1 extracellular domain
PSMA: prostate-specific membrane antigen
scDb: single chain diabody
scFab: single chain Fab
scFv: single-chain variable fragment
sctb: single chain triplebody
scTsAb: single chain trispecific antibody
SCLC: small cell lung cáncer
sdAb: single domain antibody
TAA: tumor-associated antigen
TAC: tsAb conjugate
TetraKE: tetraspecific killer engager
TriKE: trispecific killer engager
TriNKET: trispecific, NK cell engager therapies
TriTE: trispecific T-cell engager
TsAb: trispecific antibody
ULBP2: natural ligand for human NKG2D receptor
